# Determination of vitamins, minerals, and microbial loads of fortified nonalcoholic beverage (*kunun zaki*) produced from millet

**DOI:** 10.1002/fsn3.267

**Published:** 2015-07-30

**Authors:** Olusegun A. Olaoye, Stella C. Ubbor, Ebere A. Uduma

**Affiliations:** ^1^ Department of Food Science and TechnologyMichael Okpara University of AgricultureUmudikeAbia StateNigeria

**Keywords:** *Kunun zaki*, microbial loads, nutritional deficiency, sensory evaluation, tigernut milk extract

## Abstract

The objective of this study was to evaluate the possibility of fortifying *kunun zaki* with tigernut milk extract due to nutritional deficiency of the former. *Kunun zaki* and tigernut milk extract (TME) were produced using traditional methods, with little modification. They were mixed in respective percentages of 90:10 (KN10), 80:20 (KN20), and 70:30 (KN30) while whole *kunun zaki* without addition of tigernut milk extract (KN00) served as control. The resulting *kunun zaki* samples were analyzed for proximate composition, vitamins, minerals, microbial loads, and sensory evaluation. Results showed improvement in thiamine and riboflavin contents of the fortified samples over the unfortified counterparts, with the KN30 sample having highest values of 1.05 and 0.56 mg/kg thiamine and riboflavin, respectively. Minerals were higher in the samples containing TME than their KN00 counterparts; the KN30 sample had highest values of 23.5, 8.8, 148.9, 63.7, 6.7, and 18.6 mg/100 mL for respective Na, Ca, K, Mg, P, and Fe while lowest values were recorded for the KN00 sample. Microbial analysis indicated that total viable bacteria and yeast and molds were in the range 2.2–2.6 and 2.1–2.7 log CFU/g, respectively, while there was no detection of coliforms and *Staphylococcus* in the samples. The sensory evaluation of the *kunun zaki* samples indicated that higher mean scores were recorded for samples containing TME than those without it in most of the attributes tested. The KN30 sample was most preferred, having highest mean scores of 7.2, 7.8, 6.9, and 7.4 in the attributes of appearance, flavor, taste, and acceptability, respectively. The study concluded that inclusion of tigernut extract in *kunun zaki* resulted in improved nutritional and sensory qualities.

## Introduction

*Kunun zaki* is an energy‐dense beverage normally prepared from germinated cereals and is very popular in Nigeria, especially in the Northern part. The beverage is produced from either one or more of fermented millet, sorghum, guinea‐corn, and maize. *Kunun zaki* has thirst quenching properties and is therefore extensively consumed during the dry season, though consumption may be observed throughout the year (Adelekan et al. 2013).

Millet, sorghum, and maize grains are the three principal cereals from which *kunun zaki* can be produced (Adeleke et al. 2004). It is usually flavored with such spices as ginger, black pepper, and tamarind for improvement in its taste and aroma, and also to serve as purgative and cure for flatulent conditions. It is a considerably cheap beverage drink because of the ingredients used for preparation, and this makes the product readily available (Makinde and Oyeleke 2012). The process of production involves wet milling of the cereal, wet sieving, partial gelatinization of the slurry, mild fermentation, sugar addition, and bottling.

The fermentation process may last for 12–72 h (Gaffa and Ayo 2002), after which it is kept for acidification to develop. Brief fermentation, involving mainly lactic acid bacteria and yeast, usually occurs during steeping of the grains in water over 8–48 h. Wide varieties exist in the methods of preparation depending on taste, cultural norms, and habits (Abulude et al. 2006). Additives may be used in fortification of the beverage to compensate for losses during processing, or add nutrients that are either present at low level or not present at all; thus resulting in improved nutritional quality.

Tigernuts (*Cyperus esculentus*) are cultivated throughout the world including Nigeria, especially in the northern part, and other West Africa Countries like Guinea, Cote d'ivore, Cameroon, Senegal, America and other parts of the World (Belewu and Abodunrin 2006). The nuts are valued for their highly nutritious starch content, dietary fiber and carbohydrate (mono, di and polysaccharides). The nut has also been reported to be rich in sucrose (17.4–20.0%), fat (25.50%), protein (8%), and minerals such as sodium, calcium, potassium, and magnesium (Umerie and Enebeli 1997).

*Kunun zaki* is often used as a weaning food beverage for infants in Nigeria. However, since the beverage is produced majorly from cereals, it could be deficient in nutritional quality, especially protein, vitamins, and minerals, and hence supplementation with richer sources of nutrients may be required (Adelekan et al. 2013).

As a result of the low nutritional value of this beverage, research efforts are required to ensure its improvement in nutritional quality. A good approach could be the exploitation of tigernuts in complementing nutritional deficiency that is associated with *kunun zaki*. This study was therefore undertaken to incorporate tigernut milk extract into the beverage for possible nutritional improvement.

## Materials and Methods

### Source of materials

The millet grains and sweet potatoes used in this study were obtained from National Stored Products Research Institute (NSPRI), Port Harcourt, Rivers State, and National Root Crop Research Institute (NRCRI), Umudike, Abia State, respectively. Tigernuts (*Cyperus esculentus*), spices (ginger and pepper), and sugars were purchased from a local market in Umuahia Township, Abia State, Nigeria.

### Production of *kunun zaki*

**Figure 1:**
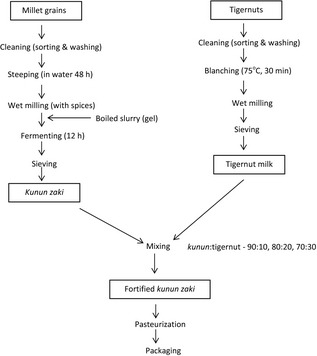
Flowchart for production of fortified *kunun zaki*.

The modified method of Ayo et al. (2013) was adopted in the production of *kunun zaki* (Fig. 1). One kilogram (1 kg) of cleaned millet grains was washed and steeped in clean water for 48 h to soften the seed. The grains were washed to remove stones and wet milled along with added spices (65 g ginger, 10 g red pepper and 15 g sweet potatoes) into slurry. Two‐third of the slurry was mixed with 2500 mL of boiling water and stirred to form a gel; this was allowed to cool for 3 h. The remaining one‐third of the slurry was added to the gel, mixed with cold boiled water (1000 mL) and left open to ferment for 12 h). It was then sieved with a muslin cloth and the filtrate was sweetened with sucrose (250 g).

### Production of tigernut milk

Tigernut milk extract was produced according to the method of Odunfa and Adeyeye (1985). Dried tigernuts (1 kg) were washed and soaked in tap water (1 L) at room temperature (30°C) for three days; changing of the soaking water was observed 24 hourly. The tigenuts were washed and then boiled in 0.2% (w/v) solution of sodium bicarbonate for 30 min to reduce objectionable flavor. The nuts were drained, mixed with water (ratio 1:4) and milled (Philips kenwood, UK). The homogenous slurry was filtered using a muslin cloth and the resultant filtrate was tigernut milk (Fig. 1).

### Formulation of enriched *kunun zaki*

*Kunun zaki* and tigernut milk were mixed in three different proportions of 90:10, 80:10, and 70:30 (v/v) of the respective products, and coded as KN10, KN20, and KN30, respectively. A sample of *kunun zaki* with no addition of tigernut milk served as control (KN00). The various samples were pasteurized at 70°C for 30 min.

### Proximate analysis

Proximate analysis of moisture, ash, fat, and protein contents of kunun zaki samples was carried out using the methods of Association of Official Analytical Chemists (AOAC 2005). Carbohydrate was determined by difference.

### Physical properties and vitamin contents

Total solid was determined by evaporating 25 mL of *kunun zaki* on a boiling water bath which was followed by drying to constant weight in an oven at 130°C for 2–3 h.

$$\%\text{Total solid}=\frac{\text{Dry weight}\times 100}{\text{Weight of sample}}$$

Specific gravities of the samples were determined as described by Pearson (1976). Vitamin C, thiamine, riboflavin, and niacin were determined according to the methods of AOAC, Association of Official Analytical Chemists (2005).

### Mineral analysis

Analysis of potassium content of the samples was carried out using flame photometry, while phosphorus was determined by the phosphovanado‐molybdate method (AOAC, Association of Official Analytical Chemists 2005). The other elemental contents (Na, Ca, Mg, and Fe) were determined, after wet digestion of sample ash with an Atomic Absorption Spectrophotometer (AAS, Hitachi Z6100, Tokyo, Japan). All determinations were carried out in triplicates.

### Microbial analysis

Ten milliliter of *kunun zaki* samples was thoroughly mixed in 90 mL sterile distilled water to obtain 10^−1^ dilution, from which further dilutions were made. One milliliter of appropriate dilutions was mixed with molten medium (45°C) using potato dextrose agar (PDA) for molds; malt extract agar (MEA) supplemented with streptomycin for yeast; MacConkey agar for coliforms; Nutrient agar (NA) for total viable bacteria; and Mannitol salt agar for *Staphylococcus*. Incubation period was 48 h at 37°C except for yeast and molds (25°C, 72 h). Determinations were carried out in triplicates and counts were expressed in logarithmic of colony‐forming unit per mL of sample (log CFU/mL)

### Sensory evaluation

The *kunun zaki* samples were subjected to sensory evaluation for the attributes of appearance, viscosity, aroma, taste, and acceptability. A semitrained twenty member panel was used and scores were allocated to the attributes based on a 9‐point hedonic scale ranging from 1 (dislike extremely) to 9 (like extremely). The data collected were subjected to statistical analysis to determine possible differences among samples.

### Statistical analysis

Data which depended on different percentages of *kunun zaki* and tigernut milk extract were analyzed according to a completely randomized design with three replicates. Data were subjected to variance analyses and differences between means were evaluated by Duncan's multiple range test using SPSS statistic programme, version 10.01 (SPSS 1999). Significant differences were expressed at *P* < 0.05.

## Results and Discussion

Table 1 shows the result of proximate analysis carried out on the *kunun zaki* samples. pH values of the products were between 5.7 and 6.0. No significant differences (*P* < 0.05) were recorded in the pH values among samples. A number of the pH values recorded in this study were slightly higher than those reported in previous studies (Agarry et al. 2010; Ayo et al. 2013); probably due to slight difference in fermentation period and cereal types as well as processing conditions used in the studies. Agarry et al. (2010) recorded pH value of 5.44 in *kunun zaki* produced from millet and this is similar to those recorded in the present study. Lower values were obtained by the authors when cereal marsh was treated with cultures of lactic acid bacteria prior to fermentation. Moreover, Belewu and Abodunrin (2006) recorded a pH of about 6 which is also similar to those recorded in the present study. The pH of about 6.0 or slightly below is usually associated with the beverage as a result of slight fermentation that is associated during production. *Kunun zaki* enriched with tigernut milk extracts (TME) recorded higher protein contents than the control containing no tigernut milk extract (KN00). The highest protein content of 6.14% was observed in sample with 30%v/v TME while lowest value of 4.96 was obtained for control sample. Increase in protein content was noticed as percentage tigernut milk extract increased, suggesting that the tigernut milk extract contributed to the protein contents of *kunun zaki* samples. This is as a result of significant differences (*P* < 0.05) recorded in the protein contents of the samples containing TME in comparison to the control. The protein content of KN00 was higher than the value reported by Ayo et al. (2013) and this may be attributed to the different cereals adopted during the production of the beverage. The improved protein contents of the *kunun zaki* samples containing TME recorded in this study may be beneficial to consumers of the product as majority of people in Nigeria could not afford protein sources of foods such as meat and egg for economic reasons.

**Table 1:** Proximate analysis of the *kunun zaki* samples

*Kunun*	Proximate parameters					
	pH	MC (%)	Protein (%)	Ash (%)	Fiber (%)	Carbohydrate (%)
KN00	5.7^a ^± 0.0	87.27^b ^± 0.14	4.96^c ^± 0.10	2.09^b ^± 0.02	0.84^a ^± 0.02	3.8^a ^± 0.23
KN10	5.9^a ^± 0.0	87.71^b ^± 0.13	5.31^b ^± 0.20	2.93^ab ^± 0.02	0.81^a ^± 0.04	2.99^b ^± 0.38
KN20	6.0^a ^± 0.0	88.41^a ^± 0.12	5.72^b ^± 0.10	3.31^a ^± 0.03	0.78^a ^± 0.11	1.72^c ^± 0.12
KN30	6.0^a ^± 0.0	89.37^a ^± 0.08	6.14^a ^± 0.15	3.44^a ^± 0.02	0.75^a ^± 0.05	0.14^d ^± 0.00

MC, moisture content; KN00, unfortified *kunun zaki*; KN10, *kunun zaki* fortified with 10% tigernut milk extract; KN20 *kunun zaki* fortified with 20% tigernut milk extract; KN30, *kunun zaki* fortified with 30% tigernut milk extract.

Values are mean scores of three replicated samples. Values in columns with different superscript letters are significantly different (*P* < 0.05).

The result of the ash contents of the beverage samples was similar to those of the protein contents; ash contents increased correspondingly with increase in incorporation of TME. The highest ash content of 3.44 was observed in sample containing 30% tigernut extract while control sample had the lowest value (3.09). It is interesting to note that tigernut contributed significantly to ash contents of the beverage as a results of higher values recorded in samples containing TME than the control sample; significant differences were also noted between the samples. In a related study, Makinde and Oyeleke (2012) reported increase in the ash contents of *kunun zaki* enriched with extract of sesame seeds over the control sample without the extract. Values of the ash contents in the *kunun zaki* recorded by the research workers are similar to those obtained in the present study. However, Ogbonna et al. (2013) and Adelekan et al. (2013) obtained ash contents of higher values in their findings on *kunun zaki* than those recorded in this study. The difference could be attributed to the different types of cereals used in the production of the beverage in the different studies. Different cereal types have abilities to contribute to the ash contents of *kunun zaki* as a result of the differences in their ash compositions.

The crude fibers of the samples remain statistically insignificant (*P* > 0.05). Although values seemed to decrease with increase in incorporation of TME, however the addition of the extract did not have any significant effect. The control sample had the highest value of 0.84 compared to the lowest (0.75) obtained for sample containing 30% TME. This could be as a result of higher content of fiber contained in millet than tigernut (Belewu and Abodunrin 2006).

The physicochemical properties and vitamin contents (Table 2) of the *kunun zaki* samples indicate that total solids ranged between 10.66 and 12.31, with the control having the highest value while sample containing 30% TME had the lowest. In a study carried out by Ayo et al. (2013) on the production of *kunun zaki*, higher values of total solids were recorded than those obtained in the present study. The difference may be as a result of the difference in the recipes used during production; lower quantity of water was used by Ayo et al. (2013) than that used in the present study. A contrary observation to the trend of total solids was made for the specific gravity which ranged from 0.73 and 0.85; specific gravity increased with increase in addition of TME. Adelekan et al. (2013) recorded a similar value of about 0.75 for specific gravity in *kunun zaki* samples.

**Table 2:** Physical properties and vitamin contents of the *kunun zaki* samples

*Kunun*	Physical properties and vitamins					
	TS (%)	SG (g/cm^3^)	Vitamin C(mg/l00 g)	Thiamine (mg/kg)	Riboflavin (mg/kg)	Niacin (mg/kg)
KN00	12.31^a ^± 0.66	0.73^a ^± 0.03	18.77^a ^± 1.02	0.71^bc ^± 0.13	0.35^c ^± 0.06	1.17^a ^± 0.12
KN10	12.28^a ^± 0.13	0.77^a ^± 0.00	13.49^b ^± 1.02	0.69^c ^± 0.04	0.36^c ^± 0.05	0.45^c ^± 0.20
KN20	11.64^b ^± 0.14	0.81^a ^± 0.00	12.91^c ^± 2.02	0.78^b ^± 0.06	0.43^b ^± 0.06	0.61^b ^± 0.02
KN30	10.66^c ^± 0.08	0.85^b ^± 0.00	12.91^c ^± 1.02	1.05^a ^± 0.06	0.56^a ^± 0.06	0.59^b ^± 0.04

TS, total solids; SG, specific gravity; KN00, unfortified *kunun zaki*; KN10, *kunun zaki* fortified with 10% tigernut milk extract; KN20 *kunun zaki* fortified with 20% tigernut milk extract; KN30, *kunun zaki* fortified with 30% tigernut milk extract.

Values are mean scores of three replicated samples. Values in columns with different superscript letters are significantly different (*P* < 0.05).

Moreover, increase in the contents of thiamine and riboflavin was recorded in the *kunun zaki* containing TME (Table 2), and this may contribute to nutritional intake of consumers. The control sample had lowest values of 0.71 and 0.35 of thiamine and riboflavin while the sample with 30% TME had highest values of 1.05 and 0.56 for the respective vitamins. The reverse was, however, recorded in the values of vitamin C and niacin. The increase recorded in some of the vitamins, especially thiamine and riboflavin, in the samples could be due to incorporation TME (Bernat et al. 2014).

Analysis of mineral contents of the *kunun zaki* samples indicates that sample without TME recorded the lowest value (21.5) for sodium while the highest (23.5) was obtained for sample containing 30% TME (Table 3). Similar trends were observed for calcium, potassium, magnesium, phosphorus, and iron in the *kunun zaki* samples; the sample with 30% TME recorded highest values while the lowest was obtained for the control sample. The values recorded for the beverage samples were slightly different from those reported by Makinde and Oyeleke (2012) in *kunun zaki*; this could be due to the different cereals adopted for production of the products. Calcium and iron contents were, however, similar to those reported by Adelekan et al. (2013). The report of Ogbonna et al. (2013) also corroborates the values of magnesium and iron recorded in this study. Higher values of the minerals recorded in this study than those reported in other studies could obviously be attributed to the incorporation of TME into the *kunun zaki* beverage. The increase in the minerals contents in the samples containing TME could therefore justify the need to enrich the beverage with sources that are rich in other nutrients lacking in cereals normally adopted in its production (Bernat et al. 2014). Minerals are of great importance in diet as they play important roles in body metabolism. For example calcium helps in the regulation of muscle contractions and transmission of nerve impulses as well as bone and teeth development (Cataldo et al. 1999). Phosphorus has also been reported to be required for bone growth, kidney function, cell growth, and maintaining the body's pH balance (Fallon and Enig 2001). Furthermore, potassium is essential for its important role is the synthesis of amino acids and proteins. Moreover, magnesium helps in relaxation of the muscle and in the formation of strong bones and teeth. It also plays fundamental roles in most reactions involving phosphate transfer, believed to be essential in the structural stability of nucleic acid and intestinal absorption while its deficiency can cause severe diarrhea, hypertension, and stroke (Appel 1999). The increase in the contents of the minerals recorded in the *kunun zaki* samples containing TME could therefore be of nutritional advantage to consumers of the products.

**Table 3:** Mineral contents of the *kunun zaki* samples

*Kunun*	Minerals (mg/100 mL)					
	Na	Ca	K	Mg	P	Fe
KN00	21.5^b ^± 1.2	5.6^c ^± 1.2	140.2^b ^± 12.9	34.9^d ^± 3.2	2.1^d ^± 0.0	9.3^b ^± 5.4
KN10	22.0^b ^± 2.1	5.8^c ^± 0.9	143.8^ab ^± 3.1	44.5^c ^± 0.1	3.5^c ^± 0.2	13.8^b ^± 0.8
KN20	22.1^b ^± 0.8	6.5^b ^± 1.7	144.5^ab ^± 7.2	49.1^b ^± 1.7	4.9^b ^± 1.1	15.2^a ^± 3.2
KN30	23.5^a ^± 4.1	8.8^a ^± 1.0	148.9^a ^± 9.4	63.7^a ^± 3.8	6.7^a ^± 0.6	18.6^a ^± 2.7

KN00, unfortified *kunun zaki*; KN10, *kunun zaki* fortified with 10% tigernut milk extract; KN20 *kunun zaki* fortified with 20% tigernut milk extract; KN30, *kunun zaki* fortified with 30% tigernut milk extract.

Values are mean scores of three replicated samples. Values in columns with different superscript letters are significantly different (*P* < 0.05).

The microbial loads of the *kunun zaki* samples are presented in Table 4. The total viable bacteria were between 2.2 and 2.6 while yeast and molds ranged from 2.4 to 2.7. No coliforms and *Staphylococcus* were detected in the beverage samples. The low values of microbial counts recorded in the *kunun zaki* samples could be due to heat treatment (pasteurization) given to the products during production. The total viable counts recorded in this study were similar to those reported in findings of Ayo et al. (2013) and Adelekan et al. (2013); the authors reported average microbial loads of 3.0 and 2.5, respectively, in *kunun zaki*. The latter also reported an average fungal count of about 2.2 in the beverage and this corroborates those recorded in the present study. The nondetection of coliforms and *Staphylococcus* in the samples could be as a result of good manufacturing and hygiene practices observed during production. Coliforms are majorly of fecal origin and their presence in foods indicates contamination from fecal sources which is highly undesirable; this is because some coliforms such as *Escherichia coli* can cause diseases such as gastroenteritis, diarrhea, and urinary tract infections (Pelczar et al. 1993).

**Table 4:** Microbial loads (log CFU/g) of the *kunun zaki* samples

*Kunun*	Microbial loads			
	TVB	Y & M	Coliforms	*Staphylococcus*
KN00	2.2 ± 0.1^b^	2.7^a^	ND	ND
KN10	2.4 ± 0.9^ab^	2.1^b^	ND	ND
KN20	2.1 ± 0.6^b^	ND	ND	ND
KN30	2.6 ± 0.1^a^	2.4^b^	ND	ND

TVB, total viable bacteria; Y & M, yeast and molds; KN00, unfortified *kunun zaki*; KN10, *kunun zaki* fortified with 10% tigernut milk extract; KN20 *kunun zaki* fortified with 20% tigernut milk extract; KN30, *kunun zaki* fortified with 30% tigernut milk extract; ND, nondetectable

Values are mean scores of three replicated samples. Values in columns with different superscript letters are significantly different (*P* < 0.05).

The means scores obtained for sensory evaluation of the *kunun zaki* samples indicate that the sample containing 30% TME recorded highest scores of 7.1, 7.8, 6.9, and 7.4 in the respective attributes of appearance, flavor, taste, and acceptability (Table 5). Significant differences (*P* < 0.05) were recorded between the samples without TME and those with the extract.

**Table 5:** Sensory attributes of the *kunun zaki* samples

*Kunun*	Attributes				
	Appearance	Viscosity	Flavour	Taste	Acceptability
KN00	6.9^a ^± 0.83	7.1^b ^± 0.85	4.6^c ^± 0.11	5.1^c ^± 0.65	6.1^c ^± 0.55
KN10	6.8^a ^± 0.80	6.7^b ^± 0.85	5.9^b ^± 0.90	6.0^b ^± 0.69	6.7^b ^± 0.63
KN20	6.9^a ^± 0.83	5.9^b ^± 0.90	6.8^ab ^± 0.81	6.1^b ^± 0.55	6.7^b ^± 0.63
KN30	7.2^a ^± 0.52	5.7^a ^± 0.03	7.8^a ^± 0.75	6.9^a ^± 0.85	7.4^a ^± 0.40

KN00, unfortified *kunun zaki*; KN10, *kunun zaki* fortified with 10% tigernut milk extract; KN20 *kunun zaki* fortified with 20% tigernut milk extract; KN30, *kunun zaki* fortified with 30% tigernut milk extract.

Values are mean scores of three replicated samples. Values in columns with different superscript letters are significantly different (*P* < 0.05).

From the results of this study, it could be concluded that incorporation of tigernut milk extract into *kunun zaki* gave some degree of fortification of the product as a result of enhanced quantities of protein, ash, and vitamins. There was also improvement in the sensory quality. Inclusion of tigernut milk extract should therefore be encouraged among producers of the beverage drink as a result of the derivable nutritional benefits that consumers can gain. However, good manufacturing and good hygiene practices should be given utmost importance during production to avoid microbial contamination that may cause foodborne illness.

## Conflict of Interest

None declared.
